# Tendon grafting using Gracilis and semitendinosus tendons for extensive flexor tendon defects following synovial sarcoma excision: A case report

**DOI:** 10.1016/j.jpra.2026.09.034

**Published:** 2026-09-29

**Authors:** Kensho Suzuki, Mika Akahane, Akari Mori, Kaoru Tada, Satoru Demura

**Affiliations:** Department of Orthopaedic Surgery, Graduate School of Medical Sciences, Kanazawa University, Kanazawa, Japan

**Keywords:** Synovial sarcoma, Flexor tendon reconstruction, Gracilis tendon, Semitendinosus tendon, Hand surgery

## Abstract

Soft-tissue sarcomas of the hand are rare. Wide excision often causes functional impairment. Reconstructing extensive flexor tendon defects after tumor excision is challenging because suitable donor tendons with adequate length and diameter are limited. We report a 39-year-old female with synovial sarcoma on the volar wrist. Tumor excision resulted in multiple flexor digitorum profundus (FDP) tendon defects. We used gracilis and semitendinosus tendons from the ipsilateral lower limb for reconstruction. The gracilis tendon reconstructed the index finger FDP. The semitendinosus tendon reconstructed the middle, ring, and little finger FDP tendons. At one year postoperatively, the patient showed satisfactory functional outcomes. The Quick Disabilities of the Arm, Shoulder and Hand (QuickDASH) score was 4.5 points. This case suggests that gracilis and semitendinosus tendons are effective alternative autografts for extensive flexor tendon reconstruction. They provide adequate length and diameter with minimal donor site morbidity.

## Introduction

Soft-tissue sarcomas of the hand are uncommon and may cause significant functional impairment when treated with wide excision. Tendon grafting is necessary for reconstructing extensive flexor tendon defects following tumor excision. However, commonly used tendons such as palmaris longus and plantaris may be absent or too small for reconstruction, especially when multiple grafts are needed. We report a case using gracilis and semitendinosus tendons for flexor tendon reconstruction following synovial sarcoma excision.

### Case report

A 39-year-old female presented with a gradually enlarging, painful mass on the volar aspect of her right wrist for six months. Examination revealed a 20 mm firm mass with tenderness but no erythema or heat. Magnetic resonance imaging showed a solid mass extending from the radioulnar joint to the subcutaneous tissue. The median nerve coursed volar and radial to the tumor. The tumor stayed within the fascial plane around the FDS and FDP tendons, with no nerve invasion. (Fig. 1) Incisional biopsy and fluorescence in situ hybridization confirmed synovial sarcoma.

**Fig. 1:**
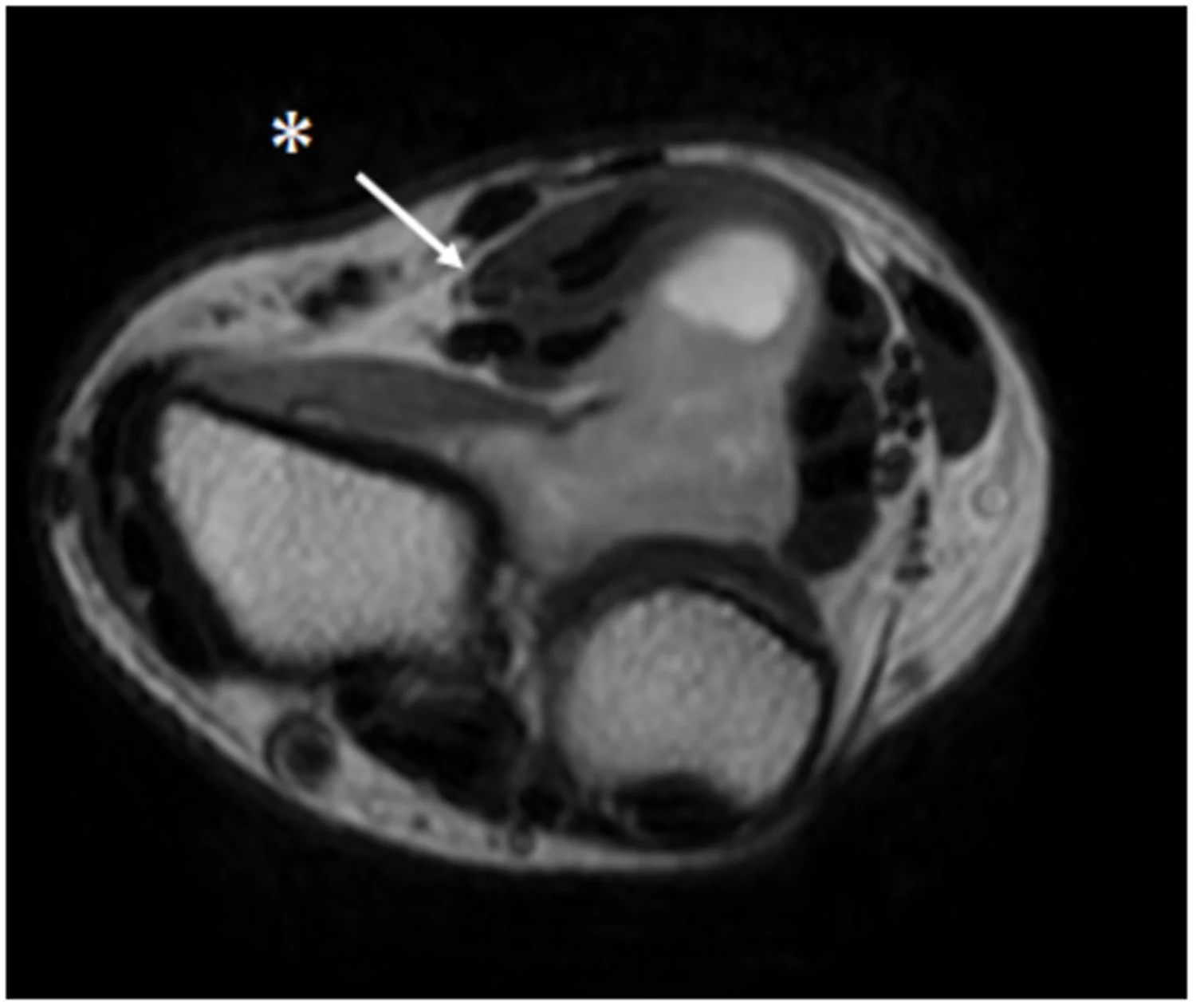
Contrast-enhanced MRI revealed a solid mass extending from the radioulnar joint space to the volar subcutaneous tissue. The median nerve (*) courses volar and radial to the tumor.

The patient received three courses of doxorubicin and ifosfamide before surgery. During surgery, the flexor carpi radialis, flexor carpi ulnaris, flexor pollicis longus, and neurovascular structures were preserved. The palmaris longus, pronator quadratus, flexor digitorum superficialis (FDS), and FDP were resected with the tumor. The FDP muscle bellies were preserved, but the tendons were resected from the musculotendinous junction to the wrist. The tumor was adherent to the periosteum and was excised with the periosteum and joint capsule. For flexor tendon reconstruction, the gracilis and semitendinosus tendons were harvested from the right lower limb. The gracilis tendon reconstructed the index finger FDP. The semitendinosus tendon was used as a single unsplit graft to reconstruct the middle, ring, and little finger FDP tendons. (Fig. 2) The A2 and A4 pulleys were preserved. The transverse carpal ligament was reconstructed after tendon grafting. Grafts were attached distally at the wrist and proximally near the musculotendinous junction. Appropriate tension was set with the wrist in neutral position, confirming the tenodesis effect.

**Fig. 2:**
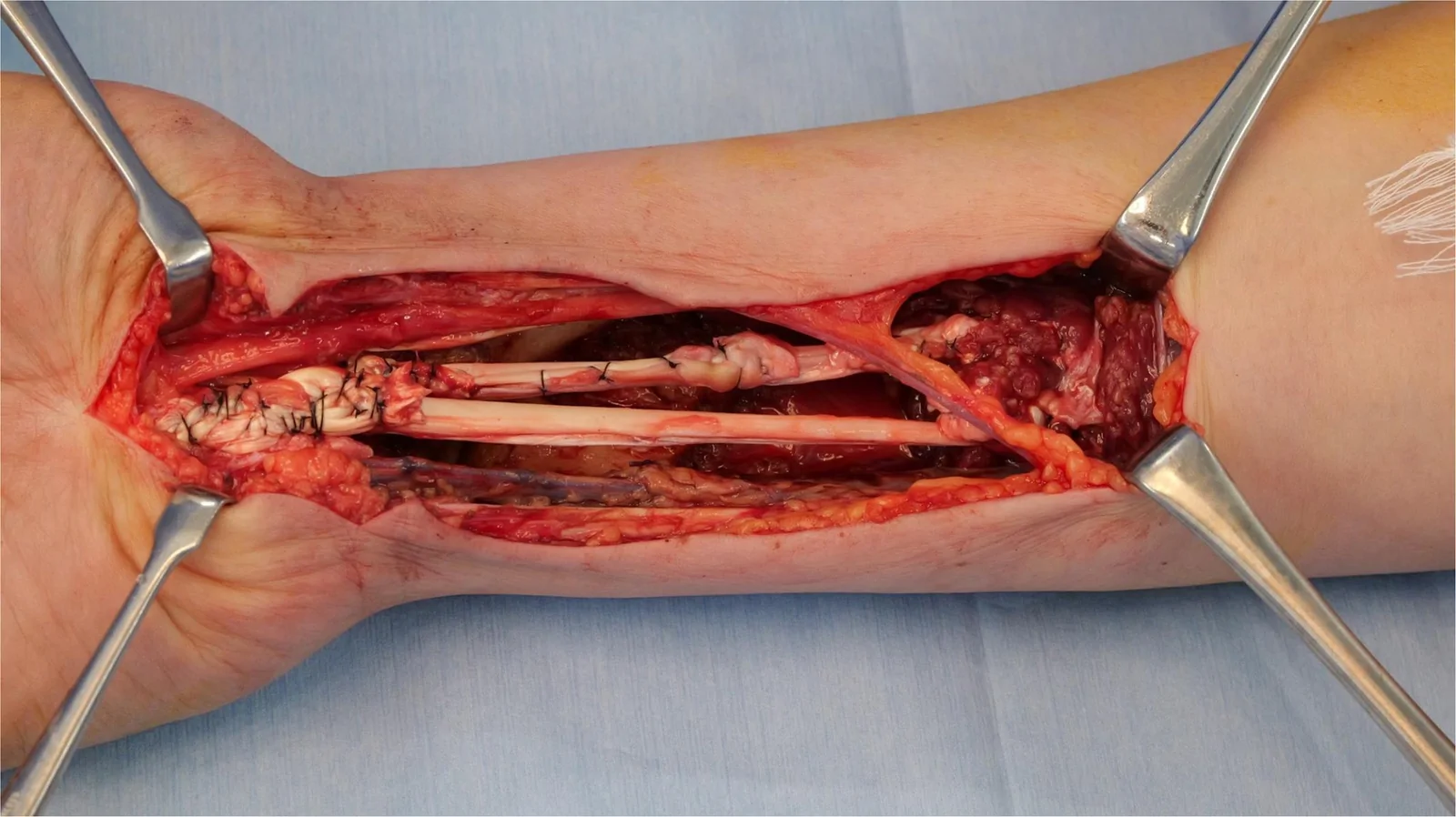
Gracilis tendon grafting was performed to the index finger FDP tendon, and semitendinosus tendon grafting was performed to the middle, ring, and little finger FDP tendons.

The tumor measured 32 × 11 × 18 mm. Pathology showed monophasic synovial sarcoma, grade 3. Wide excision achieved negative surgical margins (R0 resection). Postoperatively, active extension, passive flexion, and place-and-hold exercises were initiated immediately within a dorsal blocking splint. Active flexion exercises began at 3 weeks. At 5 weeks, the splint was removed and light activities were allowed. At 8 weeks, total pulp-to-palm distance (TPD) was 40 mm, indicating limited active flexion. A static progressive splint was introduced. At 18 weeks, TPD improved to 20–35 mm, and the static progressive splint was removed. The patient returned to work.

At one year, no tumor recurrence was observed. The median nerve remained in a similar position. (Fig. 3) Total Active Motion (TAM) measured 200 degrees for the index finger, 170 degrees for the middle finger, 165 degrees for the ring finger, and 190 degrees for the little finger, with opposition possible (Fig. 4). Grip strength was 6 kg compared to 30 kg on the unaffected side. The Quick Disabilities of the Arm, Shoulder and Hand (QuickDASH) score was 4.5 points, indicating satisfactory progress.

**Fig. 3:**
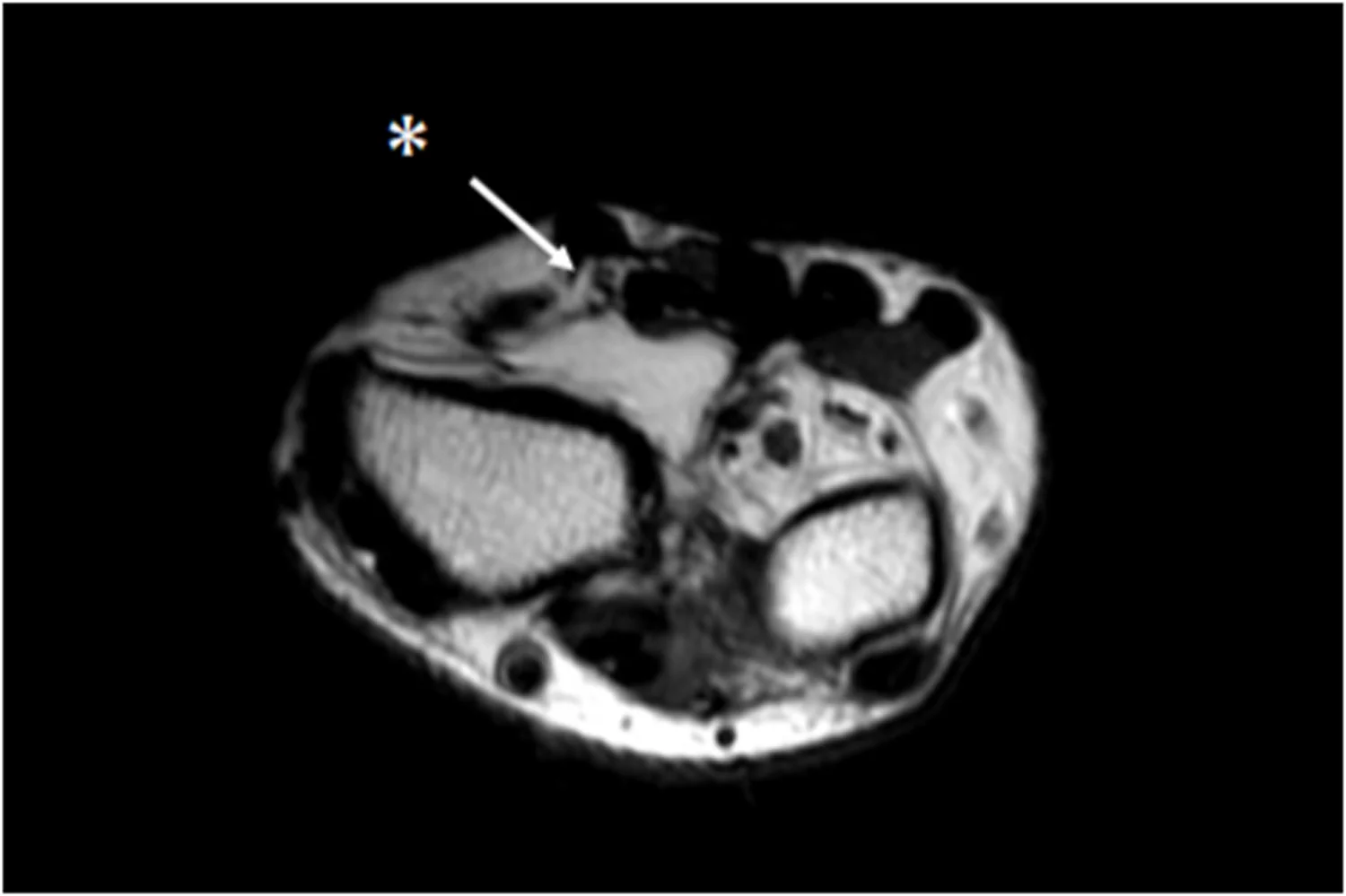
Postoperative MRI showing no evidence of tumor recurrence. The median nerve (*) remains volar and radial to the reconstructed tendon grafts, in the same position as before surgery. The white area under the nerve is fat tissue, since it shows on T1 high signal, T2 high signal, STIR low signal, and no enhancement on gadolinium-enhanced sequences. This signal pattern is most consistent with fat tissue, as the STIR sequence selectively suppresses fat signal.

**Fig. 4:**
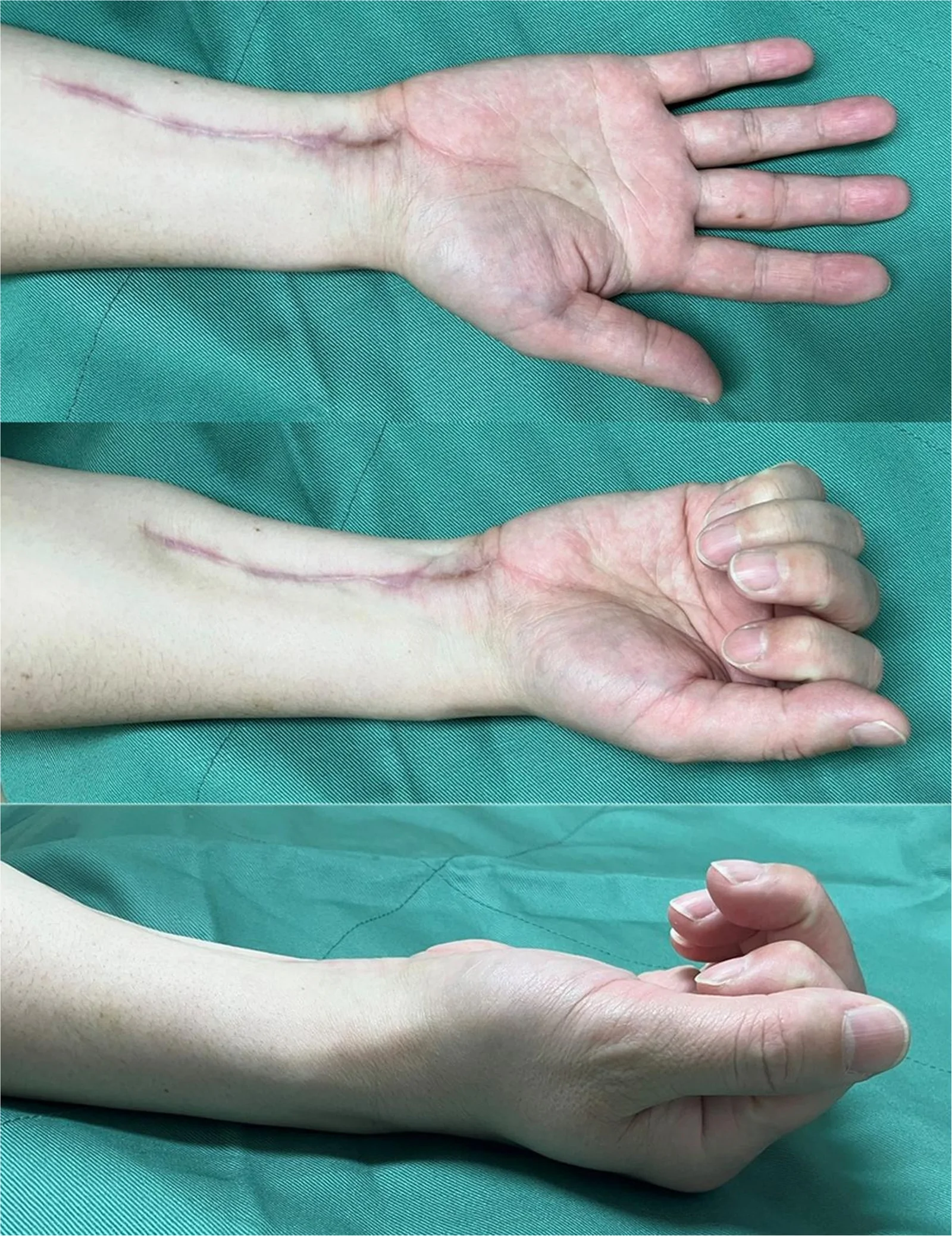
Finger flexion and extension. Range of motion showed no extension deficit with slight flexion limitation.

## Discussion

Regarding tendon graft selection, FDP tendons have diameters of 3.8–4.6 mm, and tendon grafts should ideally be at least 15 cm in length and 3 mm in diameter.^1^ Palmaris longus and plantaris tendons are commonly used as graft tendons. However, palmaris longus averages only 2.4–3.3 mm in diameter, while plantaris is even thinner at 1.9–2.3 mm.^2^ These tendons may be absent in 20.9% of forearms and 18.2% of limbs respectively,^3^ making them unsuitable for extensive reconstruction as in this case.

In contrast, gracilis averages 18.0 cm in length and 3.8 mm in diameter, while semitendinosus averages 20.0 cm and 4.8 mm.^2^ These provide sufficient dimensions with minimal donor site complications. We used gracilis for the index finger FDP due to its matching diameter and semitendinosus for the other fingers due to its larger diameter.

In a similar case report, Low et al. performed tendon grafting using semitendinosus tendons for middle and ring finger FDP tendon ruptures.^4^ TAM was 250° for the middle finger, which was generally good, but limited to 175° for the ring finger. Özbaydar et al. reported a study on multiple extensor tendon reconstruction with semitendinosus and gracilis tendon grafts and flap coverage for severe dorsal hand injuries.^5^ In their series of 10 patients who underwent single-stage reconstruction, Miller's scoring system demonstrated excellent results in 8 digits, good results in 12 digits, and fair results in 5 digits, suggesting that hamstring tendons are appropriate for primary reconstruction due to their availability and compatibility. While hamstring tendons have been used for tendon grafting, combined use of gracilis and semitendinosus tendons for flexor tendon reconstruction is rarely reported. In this case, the semitendinosus tendon was used as a single unsplit graft attached to three FDP tendons simultaneously, reconstructing the middle, ring, and little fingers. Additionally, simultaneous reconstruction of multiple FDP tendons following oncologic resection is extremely limited in the literature. This case demonstrates that combined use of gracilis and semitendinosus tendons is an effective option for extensive flexor tendon defects.

Our case achieved favorable outcomes with the QuickDASH score of 4.5 points, suggesting that FDP reconstruction using gracilis and semitendinosus tendons may be effective for extensive flexor tendon defects. Grip strength recovered to only 20% of the contralateral side, measuring 6 kg compared to 30 kg. This limitation likely resulted from resection of the FDP and FDS tendons, postoperative adhesion, incomplete muscle strength recovery, and residual finger flexion limitation preventing complete grip closure. The hamstring tendons provided adequate length and diameter with minimal donor site morbidity. This approach warrants consideration for complex flexor tendon reconstruction when conventional grafts are insufficient.

This study has several limitations. First, since the semitendinosus tendon was used as a single graft attached to the middle, ring, and little finger FDP tendons, these three digits do not flex independently. Second, the follow-up period of one year is relatively short for synovial sarcoma, which can cause late local recurrence and distant metastasis. Long-term follow-up is needed to evaluate oncologic outcomes.

## Statement of informed consent

Written informed consent was obtained from the patient for publication of this case report and any accompanying images. The consent form is retained by the authors.

## Statement of human and animal rights

This case report was conducted in accordance with the principles of the Declaration of Helsinki. Our institution does not require institutional review board approval for individual case reports as this does not constitute research involving human subjects under our institutional guidelines.

## Statement of funding

The authors received no financial support for the research, authorship, and/or publication of this article.

## CRediT authorship contribution statement

**Kensho Suzuki:** Conceptualization, Investigation, Writing – original draft. **Mika Akahane:** Supervision, Writing – review & editing. **Akari Mori:** Writing – review & editing. **Kaoru Tada:** Supervision, Writing – review & editing. **Satoru Demura:** Supervision, Writing – review & editing.

## Declaration of competing interest

The authors declared no potential conflicts of interest with respect to the research, authorship, and/or publication of this article.
